# Novel insight into prediction model for sleep quality among college students: a LASSO-derived sleep evaluation

**DOI:** 10.3389/fpsyt.2025.1585732

**Published:** 2025-04-25

**Authors:** Ling Yao, Qingquan Chen, Kang Yang, Zhihua Zheng, Zhihan Chen, Danna Wang, Yining Xia, Dingquan Chen, Lufeng Chen

**Affiliations:** ^1^ The Second Affiliated Hospital of Fujian Medical University, Quanzhou, Fujian, China; ^2^ The Graduate School of Fujian Medical University, Fuzhou, Fujian, China; ^3^ The School of Public Health, Fujian Medical University, Fuzhou, Fujian, China; ^4^ Fujian Medical University, Fuzhou, Fujian, China

**Keywords:** sleep quality, college students, machine learning, LASSO regression, PSQI, ANN, prediction model

## Abstract

**Background:**

Sleep disturbance has become a significant concern among college students, as it can lead to various mental and physical disorders. This study aims to provide a fresh perspective by developing and validating a predictive model for sleep quality among college students.

**Methods:**

Data from 20,645 college students in Fujian Province, China, collected between 5th April and 16th April 2022, were analyzed. Participants completed the Pittsburgh Sleep Quality Index (PSQI) scale, a self-designed general data questionnaire, and a sleep quality influencing factor questionnaire. Multinomial logistic regression, LASSO regression, and Boruta feature selection methods were utilized to select relevant variables. The data were then divided into a training–testing set (70%) and an independent validation set (30%) using stratified sampling. Six machine learning techniques, including artificial neural network (ANN), decision tree, gradient-boosting tree, k-nearest neighbor, naïve Bayes, and random forest, were developed and validated. Finally, an online sleep evaluation website was established based on the best-fitting prediction model.

**Results:**

The mean global PSQI score was 6.02 ± 3.112, with a sleep disturbance prevalence of 28.9% (defined as a global PSQI score > 7). The LASSO regression model identified eight predictors: age, specialty, respiratory history, coffee consumption, staying up late, prolonged online activity, sudden changes, and impatient closed-loop management. Among the evaluated models, the ANN demonstrated superior performance with an area under the receiver operating characteristic curve (AUC) of 0.713 (95% CI: 0.696–0.730), accuracy of 0.669 (95% CI: 0.669–0.669), sensitivity of 0.682 (95% CI: 0.699–0.665), specificity of 0.637 (95% CI: 0.665–0.610). Decision curve analysis and clinical impact analysis further confirmed the model’s clinical utility.

**Conclusions:**

This study developed a prediction model for sleep disturbance among college students using a LASSO regression and ANN, incorporating eight predictors. The model can serve as an intuitive and practical tool for predicting sleep quality and supporting effective management and healthcare on college campuses.

## Introduction

### Sleep quality among college students

Sleep disturbances represent a major health issue that encompasses a wide range of sleep complaints, such as difficulty initiating sleep (DIS), difficulty maintaining sleep (DMS) (1), early-morning awakening (EMA) (2), non-restorative sleep (NRS), and poor sleep quality (3, 4). In addition, poor sleep quality is associated with certain medical conditions (e.g., fibromyalgia (5), arthritis/rheumatism (6, 7), heart disease, and cancer (8, 9).

The characteristics of sleep among college students are different from the general public. College students report an average of 7–7.5 hours of sleep per night, which is 1–1.5 hours fewer than their self-reported ideal of 8.5 hours per night, according to studies that suggest they suffer from chronic sleep deprivation (10). The most common sleep disorders seen in college students are inadequate sleep hygiene (ISH), delayed sleep phase disorder (DSPD), and insomnia (11). Often, university students who are transitioning from adolescence to adulthood experience numerous challenges, such as having to adapt to new social situations, leaving home, and coping with high academic and social pressures and erratic living schedules, all of which could increase the risk of sleep disturbances (12). A meta analysis calculated 14 studies (*n*=22,297) and the pooled sleep disturbance prevalence of college students is 33% (95% CI: 22-44%) (13). The delivery of behavioral sleep medicine is particularly relevant for the college student population, as the early intervention on their sleep problems might prevent lifelong consequences.

### Previous studies

Previous studies found varied rates of sleep disturbances (2) in this population, and a large proportion of them focused on specific variables that related to later adverse outcomes. For instance, chronic diseases, stressful issues (e.g., anxiety, lockdown, graduation) (14), sleep attitudes (15), lifestyles (e.g., exercise, midday rest) (16, 17), electronic device use (18), diet, and consumption of alcohol, cigarettes, drugs, and coffee were found to relate to sleep disturbances (19).

Furthermore, only a few studies have used these variables to predict sleep quality among college students. Nonetheless, despite studies that predicted sleep quality for medical staff (20), elderly patients (21), infants (22), adolescents (23), and children (24), a few models set college students as target patients, which provided a completely new insight on sleep quality predictions for campus students.

In a previous study conducted on the same population (25), 11 variables related to sleep quality were used as parameters for prediction models. Among these variables, residence had a significant impact on the sleep quality of college students. However, it was found that the place of residence could pose a challenge for migrating the model to other locations, affecting the simplicity and generalizability of the model.

### LASSO algorithm and application

The ordinary least squares method is often used by researchers to explore the relationships between variables in previous studies. However, when many predictors are included, it leads to model–data overfit and multi-collinearity.

The LASSO algorithm was first proposed by Robert Tibshirani in 1997 and is known as the least absolute shrinkage and selection operator (26). It can obtain a more refined model by constructing a penalty function that aids in compressing some coefficients while setting other coefficients to zero. Thus, it retains the advantage of subset shrinkage and is a biased estimator when dealing with data with complex covariance. Research has shown that the LASSO method is capable of constructing a more compressed model that offers greater prediction accuracy compared to other existing methods (27, 28).

The LASSO method has been utilized to provide clinical information for performing early identification of S-COVID-19-P on admissions in fever clinics with a 100% recall score, and its model has been deployed as an online triage tool (29). In addition, it performed well in selecting features to improve the mortality predictions of hospitalized patients with COVID-19 with electronic health records (30) and predict the outcomes of SARS-CoV-2 pneumonia patients based on laboratory findings (31).

In comparison to a previous study (26), which utilized a multivariate unconditional LR analysis to determine predictors, LASSO is a tool that selects fewer parameters and guarantees greater prediction accuracy, making it advantageous in applications. The LASSO method can be used as a suitable alternative machine learning technique for exploring the key predictors that affect sleep quality among college students.

### Aims

The aim of this study was to investigate the most common potential risk factors associated with poor sleep quality and further develop and validate a LASSO-derived prediction model to measure the risks of poor sleep quality among university students.

This study hypothesized that the significant variables associated with poor sleep quality could be identified and used to create an easy-to-operate website that would accurately and individually evaluate the probability of suffering from poor sleep, especially among university students.

We hope that this website will be an intuitive and practical tool for sleep quality predictions that will support early prevention in colleges, enhance more personalized and precise medical aids in hospitals, and assist in allocating appropriate health resources for governments and societies.

## Methods

### Aim and design

**Figure 1:**
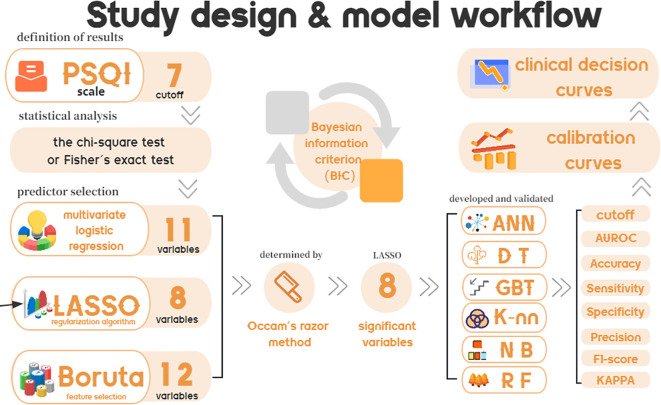
Study design and model workflow.

The objective of this study was to explore prevalent risk factors linked to impaired sleep quality among university students and subsequently develop and validate a LASSO-derived prediction model to assess the risk of poor sleep quality. **Figure 1**. shows the study design and model workflow of this study. And we ensured the security of the data.

### Setting and participants

An internet-based cross-sectional sleep quality survey was conducted for 33 universities in Fujian Province. We collected data from 23,572 full-time undergraduate or graduate students (with an age range of 17–35 years) in Fujian Province who underwent an internet-based cross-sectional sleep quality survey between April 5 and 16, 2022. Full-time undergraduate and graduate students who delayed enrollment due to the epidemic, lived outside of a student residence, or had significant sleep or mental disorders were excluded from the study.

### Scales and questionnaires

The Pittsburgh Sleep Quality Index (PSQI) is a self-rated questionnaire which assesses sleep quality and disturbances over a 1-month time interval, It is one of the most extensively used and useful tools for assessing sleep disorders. Its clinical and clinemetric qualities point to potential use in mental clinical practice and research of college students (32). Higher sleep scores on the PSQI scale equate to poorer sleep quality.

A self-designed general data questionnaire and a sleep quality influencing factor questionnaire include age, gender, residence, specialty (medical-related majors, science and engineering, or liberal arts), grade (graduating or non-Graduating), Body Mass Index (BMI), with or without respiratory history, the frequency of coffee consumption, staying up late, spending long hours online, suffering sudden changes, fearing infection of COVID-19, feeling impatient with closed-loop management (**Supplementary Text S1**).

### Definition of the results

In this study, a PSQI score of 7 was used as the cut-off point for sample grouping, with poor sleep being defined as a PSQI of > 7 and good sleep quality being defined as a PSQI of ≤ 7.

### Statistical analysis

The qualitative data were expressed as numbers and percentages and compared using a chi-square test or a Fisher’s exact test. A *p* value of < 0.05 in the univariate analysis and a *p* value of < 0.01 in the multivariate analysis were considered statistically significant. The questionnaires included in the study contained no missing data.

### Predictor selection

We used three algorithms to select predictors in the dataset. First, predictors with a *p* value of < 0.10 from the univariate analysis were entered into a multivariate logistic regression. Second, a LASSO algorithm was used for a 10-fold cross-validation to select potential predictors with non-zero coefficients. Third, the Boruta feature selection was used to identify key categorical variables. The performance of these methods was assessed against the following: Nagelkerke R^2^ (larger values are better), root-mean-square error (RMSE; lower values are better), and Bayesian information criterion (BIC; lower values are better). Finally, the optimal predictor selection algorithm was determined based on Occam’s razor.

### Prediction model development and validation

Six prediction models were built using artificial neural network (ANN), decision tree (DT), gradient-boosting tree (GBT), k-nearest neighbor (K-nn), naïve Bayes (NB), and random forest (RF). The incorporated data were divided into a training–testing set (70%) and an independent validation set (30%) using stratified sampling. To avoid overfitting and promote the models, we used a 10-fold cross-validation for the training–testing set and referenced the best models to the independent validation set. We evaluated the model’s performance by calculating the area under the receiver operating curve (AUROC) for the six models in the independent validation set, and, in addition, we calculated the accuracy, sensitivity, specificity, precision, F1-score, and KAPPA to further evaluate the model’s performance. In this study, a calibration curve analysis was performed to assess the agreement by the slope of the calibration curve (an ideal value of 1), intercept, and Brier score (an ideal value of 0; a value of >0.3 indicates poor calibration).

A decision curve analysis was performed by quantifying the net clinical benefit at different threshold probabilities, and a clinical impact curve analysis was performed by quantifying the cost–benefit ratio at different threshold probabilities to determine the clinical usefulness of the prediction model.

All machine learning models were developed and validated using R, version 4.2.1.

### Clinical applications

Prediction models have traditionally been assessed using sensitivity and specificity statistics, but these results are silent on if using the model in clinical practice would be advantageous or disadvantageous.

In this study, we utilized calibration curve analysis, decision curve analysis, clinical impact curve analysis, and net clinical benefit to compare the clinical practice performance of six models.

The calibration curve assesses the agreement between predicted probabilities and actual observations. The baseline is typically an ideal 45-degree diagonal line, representing perfect calibration where predicted probabilities equal observed probabilities. The decision curve analysis curve evaluates the clinical utility of a prediction model across different probability thresholds. The baseline represents the net benefit without the model, avoiding any benefit or harm from predictions. The clinical impact curve assesses the impact of a model’s predictions on patient management across different thresholds. Net clinical benefit is useful for determining whether basing clinical decisions on a model would do more good than harm. This is in contrast to traditional measures such as sensitivity, specificity, or area under the curve, which are statistical abstractions not directly informative about clinical value. Estimating net clinical benefit makes possible to clarify the basis for therapeutic decisions on an individual and collective level.

Net clinical benefit is defined as (33):


$$Net\;benefit=\frac{True\;positives}{n}-\frac{False\;positives}{n}(\frac{P}{1-p})$$

*n* is the total number of patients in the study and *p* is the threshold probability.

In addition, we also developed an easy-to-operate website to put model into practice. In reality, many college students refrain from visiting the hospital for a sleep quality assessment due to the inconvenience of attending an appointment. Thus, we provided a simple application for predicting the sleep quality of college students. College students who consider themselves in need of a simple screening for poor sleep quality can access the online website we created and enter the appropriate predictors into the website, which will generate predictions in real time to assist participants in making medical decisions.

## Results

### Participant characteristics

**Figure 2:**
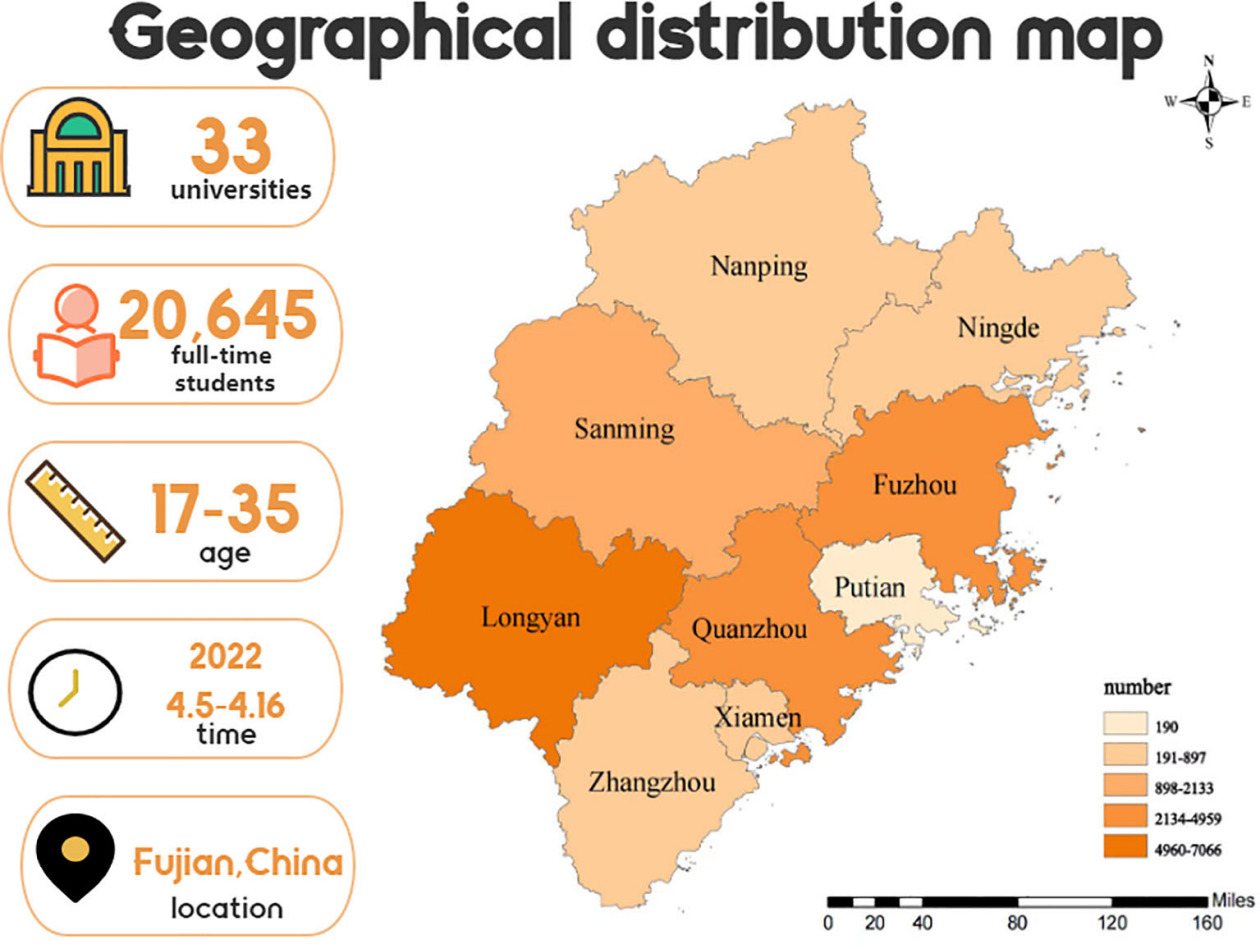
Geographical distribution map of the 20,645 college students in Fujian province between April 5 and 16, 2022.

From the included participants, the mean global PSQI score was 6.02 ± 3.112 and 14,673 had good sleep quality (71.1%) and 5,972 had poor sleep quality (28.9%). **Table 1** demonstrates a statistical analysis of the effect of different factors on sleep quality among college students. The distribution of the participants in this study covered the entire province of Fujian in China, as illustrated in **Figure 2**.

**Table 1:** Analysis of factors affecting sleep quality among college students in this survey.

Variables	*n*=20645	Sleep Quality		Univariate analysis		Multivariate analysis	*p*
		Good(≤7)	Poor(>7)	*χ^2^*	*p*	OR [95% CI]	
Age							
<20	9227	6819 (46.5%)	2408 (40.3%)	64.732	<0.001	1.00 [Reference]	<0.001
≥20	11418	7854 (53.5%)	3564 (59.7%)			1.31 [1.23, 1.40]
Gender							
Male	6326	4747 (32.4%)	1579 (26.4%)	69.801	<0.001	1.00 [Reference]	<0.001
Female	14319	9926 (67.6%)	4393 (73.6%)			1.14 [1.06, 1.23]
Residence							
Quanzhou	4959	3502 (23.9%)	1457 (24.4%)	114.574	<0.001	1.00 [Reference]	
Fuzhou	3782	2519 (17.2%)	1263 (21.1%)			0.92 [0.83, 1.02]	0.109
Longyan	7066	5293 (36.1%)	1773 (29.7%)			0.71 [0.64, 0.78]	<0.001
Nanping	897	655 (4.5%)	242 (4.1%)			0.91 [0.77, 1.09]	0.313
Ningde	540	392 (2.7%)	148(2.5%)			0.83 [0.67, 1.03]	0.091
Putian	190	146(1.0%)	44(0.7%)			0.78 [0.54, 1.11]	0.172
Sanming	2133	1451 (9.9%)	682 (11.4%)			0.93 [0.82, 1.06]	0.293
Xiamen	437	291 (2.0%)	146 (2.4%)			0.93 [0.74, 1.16]	0.524
Zhangzhou	641	424 (2.9%)	217 (3.6%)			1.57 [1.29, 1.90]	<0.001
Specialty							
Medical-related majors	4088	3120 (21.3%)	968 (16.2%)	163.534	<0.001	1.00 [Reference]	
Science and engineering	7876	5780 (39.4%)	2096 (35.1%)			1.42 [1.27, 1.58]	<0.001
Liberal arts	8681	5773 (39.3%)	2908 (48.7%)			1.62 [1.46, 1.80]	<0.001
Grade							
Graduating class	1459	998 (6.8%)	461 (7.7%)	5.443	0.021	1.00 [Reference]	0.628
Non-graduating class	19186	13675 (92.3%)	5511 (92.3%)			1.03 [0.91, 1.17]
BMI							
<18.5	4804	3381(23.0%)	1423(23.8%)	5.127	0.163		
[18.5,24)	12513	8960(61.1%)	3553(59.5%)				
[24,28)	2303	1604(10.9%)	699(11.7%)				
≥28	1025	728(5.0%)	297(5.0%)				
Respiratory history							
No	15477	11342 (77.3%)	4135 (69.2%)	146.882	<0.001	1.00 [Reference]	
Yes	5168	3331 (22.7%)	1837 (30.8%)			1.35 [1.25, 1.45]	<0.001
Coffee consumption							
No	10290	7884 (53.7%)	2406 (40.3%)	415.938	<0.001	1.00 [Reference]	
Occasionally	8331	5652 (38.5%)	2679 (44.9%)			1.18 [1.10, 1.27]	<0.001
Often	1441	799(5.4%)	642 (10.8%)			1.55 [1.37, 1.76]	<0.001
Almost everyday	583	338 (2.3%)	245(4.1%)			1.29 [1.07, 1.56]	0.007
Staying up late							
Not matched	10951	8519 (58.1%)	2432 (40.7%)	780.661	<0.001	1.00 [Reference]	
Sometimes matched	7738	5195 (35.4%)	2543 (42.6%)			1.35 [1.26, 1.45]	<0.001
Often matched	1442	746 (5.1%)	696 (11.7%)			1.93 [1.71, 2.18]	<0.001
Always matched	514	213(1.5%)	301(5.0%)			2.24 [1.84, 2.73]	<0.001
Long hours online							
Not matched	5892	4838(33.0%)	1054 (17.6%)	986.397	<0.001	1.00 [Reference]	
Sometimes matched	8915	6483 (44.2%)	2432 (40.7%)			1.29 [1.18, 1.41]	<0.001
Often matched	4005	2457 (16.7%)	1548 (25.9%)			1.83 [1.66, 2.03]	<0.001
Always matched	1833	895 (6.1%)	938 (15.7%)			2.67 [2.36, 3.02]	<0.001
Sudden changes							
No	19792	14207 (96.8%)	5585 (93.5%)	117.000	<0.001	1.00 [Reference]	
Yes	853	466 (3.2%)	387 (6.5%)			1.89 [1.63, 2.20]	<0.001
Fears of infection							
Not matched	8038	5922 (40.4%)	2116 (35.4%)	120.260	<0.001	1.00 [Reference]	
Sometimes matched	10274	7302 (49.8%)	2972 (49.8%)			0.98 [0.92, 1.06]	0.641
Often matched	1673	1055 (7.2%)	618 (10.3%)			1.19 [1.05, 1.34]	0.005
Always matched	660	394 (2.7%)	266(4.5%)			1.25 [1.04, 1.49]	0.015
Impatient closed-loop management							
Not matched	7663	6250 (42.6%)	1413 (23.7%)	1037.383	<0.001	1.00 [Reference]	
Sometimes matched	8944	6252 (42.6%)	2692 (45.1%)			1.59 [1.47, 1.72]	<0.001
Often matched	2252	1293 (8.8%)	959 (16.1%)			2.40 [2.15, 2.67]	<0.001
Always matched	1786	878(6.0%)	908 (15.2%)			3.06 [2.72, 3.45]	<0.001

OR, odds ratio; CI, confidence interval; BMI, Body Mass Index.

### Prediction feature selection

Univariate and multivariate ordered logistic regressions were used to assess the variables associated with sleep quality among college students (**Table 1**). The multivariate analysis identified 11 candidate predictors.

**Figure 3:**
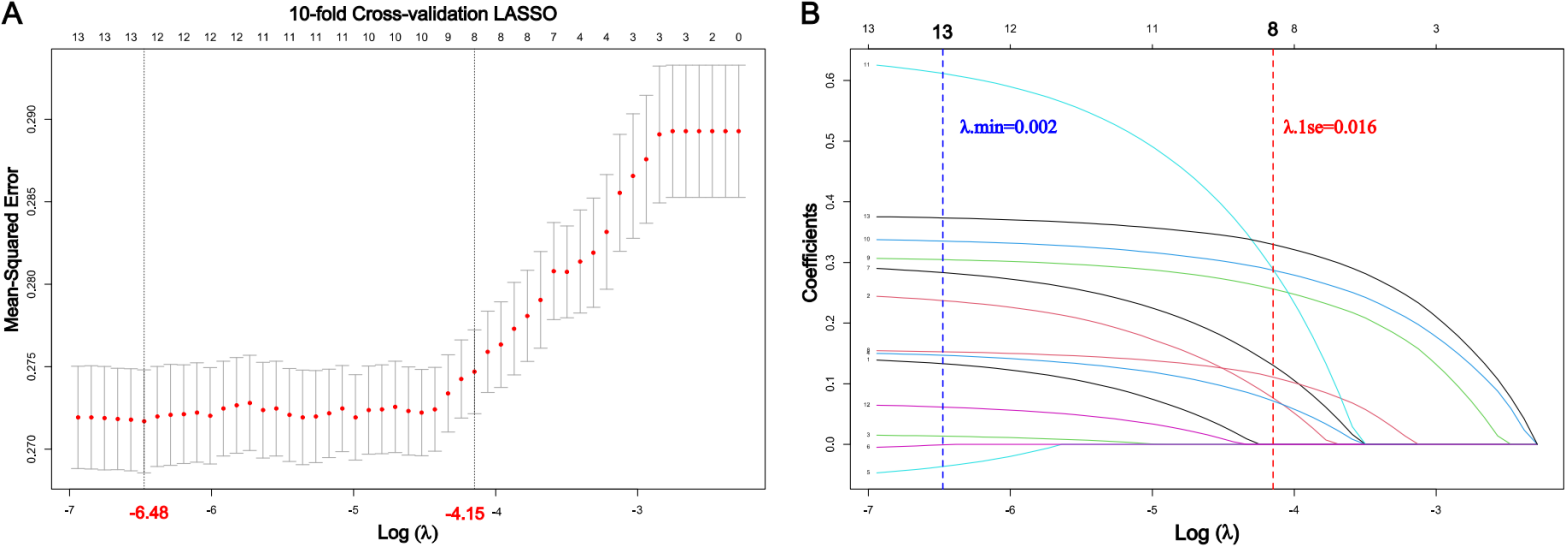
Feature selection using least absolute shrinkage and selection operator (LASSO) regression. **(A)** A LASSO-based ordinal logistic model with a 10-fold cross-validation based on the minimum mean squared error (MSE) was employed to find the optimal parameter (λ). The MSE vs. log (λ) is shown in the plot. The two dotted vertical lines indicate the optimal λ values based on the criterion of minimal MSE (λmin) or the criterion of one standard error of the minimum (λ1se). **(B)** A LASSO coefficient profile of all feature variables against the log (λ) sequence. The blue and red dotted vertical lines represent the log(λmin) and log(λ1se), respectively.

**Figure 3A** shows the results for the 13 variables included in the LASSO algorithm. When the λ value was increased to 0.016 (one standard error of the minimum value of λ), only eight candidate predictors were retained in the model, which were presumably the most influential predictors of sleep quality among college students (**Figure 3B**).

**Figure 4:**
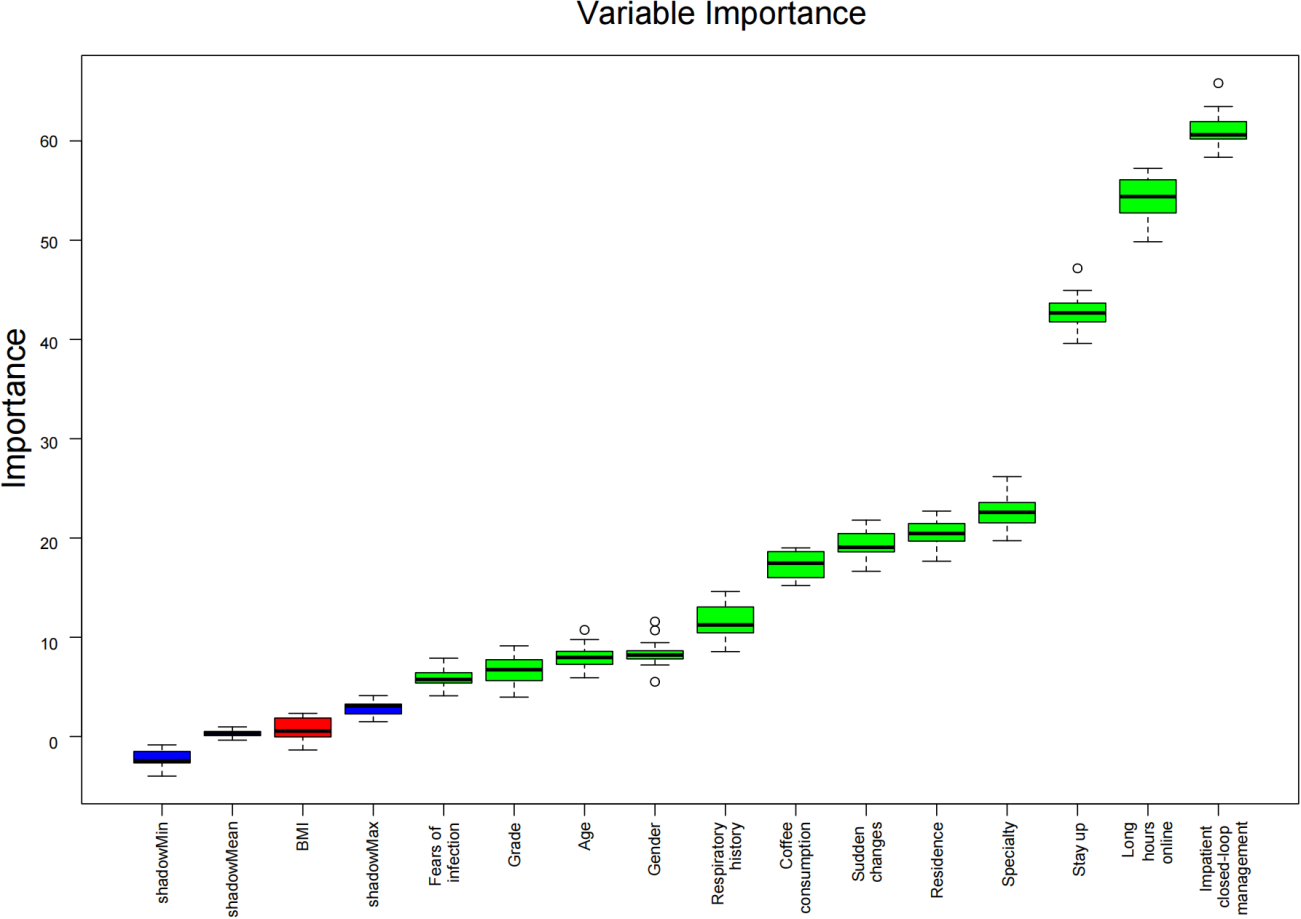
Feature selection based on the Boruta algorithm. The horizontal axis represents the name of each variable, and the vertical axis shows the Z-value of each variable. The box plot shows the Z-value of each variable during the model calculations. The green boxes represent the first 12 important variables, and the red box represents the non-important variables.

The Boruta feature selection was used to identify key categorical variables, i.e., to statistically compare the importance of the feature variables that were actually present in the data with those that were randomly added. Finally, 12 candidate predictors were identified as important variables (**Figure 4**).

LASSO can compress the coefficients of some unimportant features to zero, thereby selecting the most relevant features. Boruta is a feature selection method based on random forests, which generates a large number of candidate features and may retain some redundant or unimportant features, leading to a higher model complexity. In our study, LASSO selected 8 of the most important predictor variables through 10-fold cross-validation, and these variables demonstrated high predictive accuracy (AUROC of 0.713) in subsequent models such as artificial neural networks (ANN). Although Boruta also identified 12 important features, the features selected by LASSO performed better in terms of model prediction performance. Compared with Boruta, the LASSO regularization algorithm was identified as the optimal predictor selection algorithm based on Occam’s razor.

LASSO introduces sparsity through L1 regularization and performs automatic feature selection. Compared with other regularization methods, unlike Ridge regression (L2 regularization), which shrinks all coefficients but does not exclude any features, LASSO tends to produce sparse solutions by completely eliminating irrelevant features. While Elastic Net combines L1 and L2 regularization, it is computationally more complex in high-dimensional settings. LASSO strikes an optimal balance between computational efficiency and sparsity, making it a preferred choice for this study.

**Figure 5:**
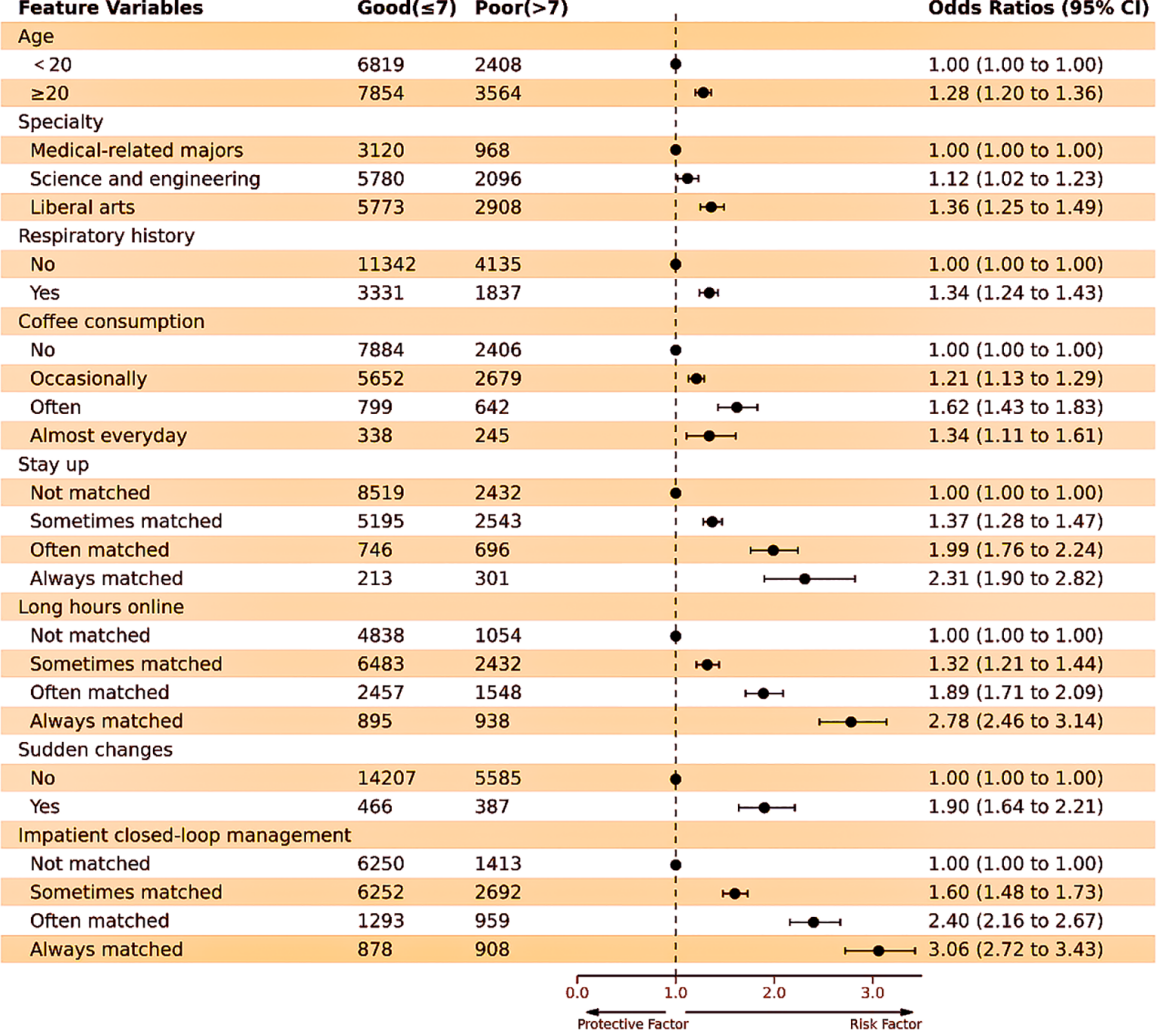
Forest plot of ORs for 8 predictors included in the prediction model. The black dots and horizontal lines correspond to the OR and 95% CI values. ORs with 95% CIs are shown on the right-hand side of the figure.

The final predictors included in the prediction model were as follows: age, specialty, respiratory history, coffee consumption, staying up late, long hours online, sudden changes, and impatient closed-loop management. **Table 2** represents the performance of three feature selection methods. The OR and 95% CI values of the included predictors are shown in **Figure 5**.

**Table 2:** Performance of three feature selection methods.

Feature Selection Method	No. of Feature Variables	AUROC (95% CI)	Accuracy (95% CI)	Nagelkerke R^2^	RMSE	BIC
Univariate and multivariate stepwise regression	11	0.704(0.697-0.712)	0.670(0.670-0.670)	0.091	0.428	22705.47
LASSO regression	8	0.700(0.693-0.708)	0.676(0.676-0.676)	0.100	0.430	-34694.65
Boruta and stepwise regression	12	0.704(0.697-0.712)	0.678(0.678-0.678)	0.091	0.428	22714.36

*AUROC*, the area under the receiver operating curve; *RMSE*, root-mean-square error; *BIC*, Bayesian information criterion.

### Development and validation of a sleep quality prediction model for college students

**Figure 6:**
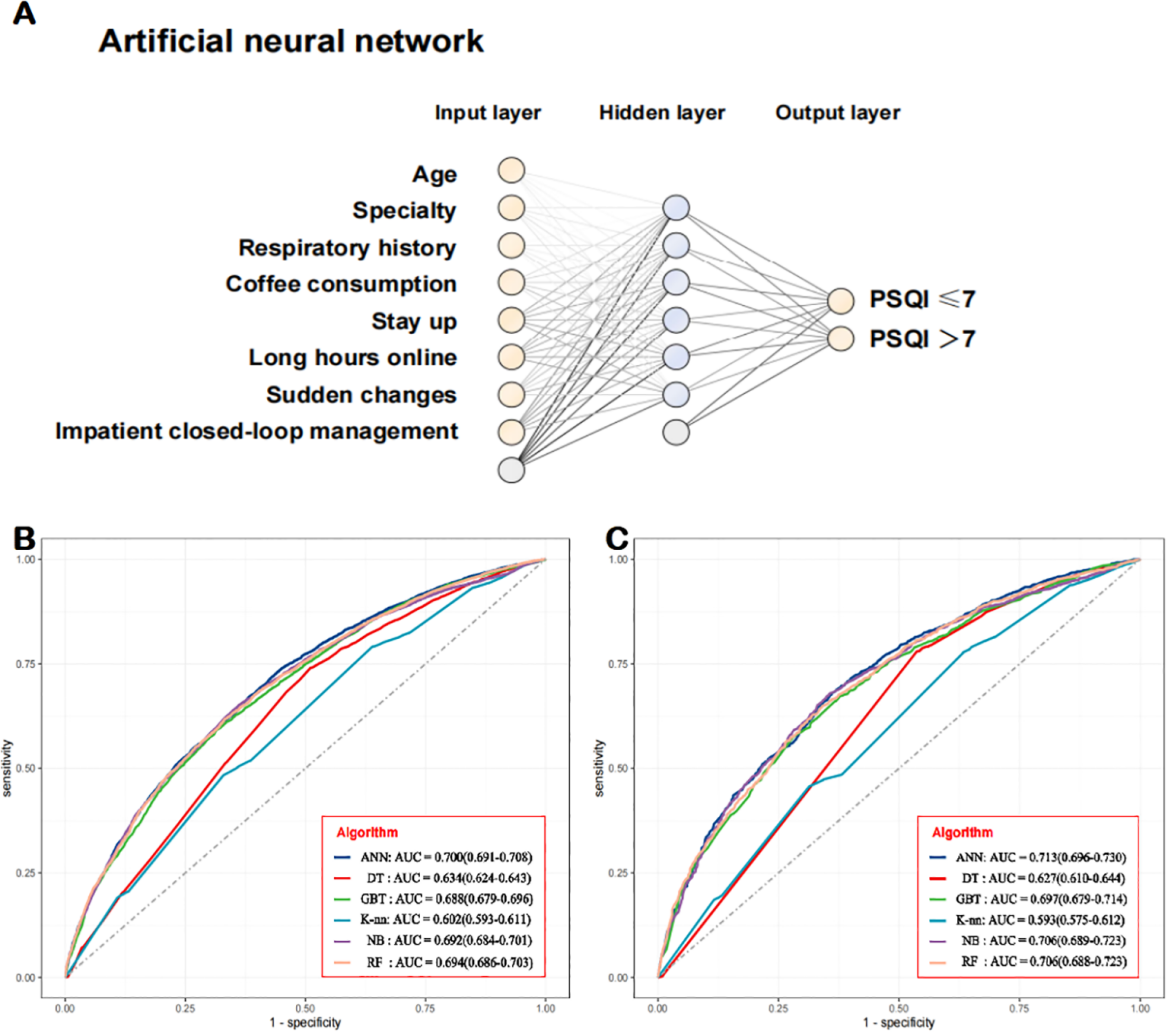
Development and application of a model for predicting the risk of a PSQI score of > 7. The prediction model. **(B, C)** Receiving operating characteristic curves showing the performance of the model in predicting sleep quality among college students in the **(B)** training–testing set and **(C)** independent validation set. .

**Figure 7:**
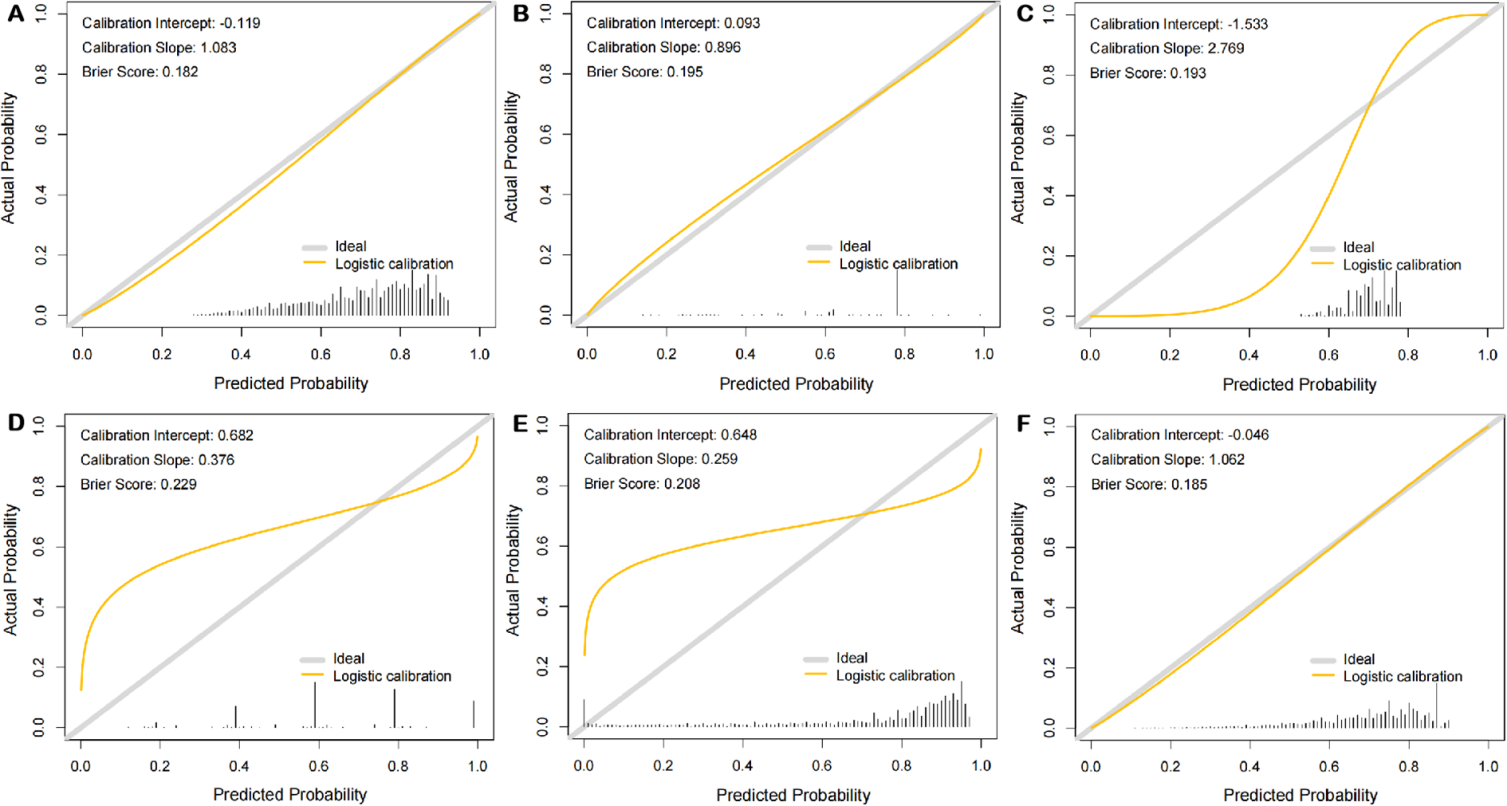
Calibration curves for testing the stability of six prediction models in the cohorts. **(A)** ANN; **(B)** DT; **(C)** GBT; **(D)** K-nn; **(E)** NB; **(F)** RF.

Finally, the eight predictors were integrated into the sleep quality risk prediction model for college students (**Figure 6A**). In the training–testing set, the AUROC values of ANN, DT, GBT, K-nn, NB, and RF were 0.700 (95% CI: 0.691-0.708), 0.634 (95% CI: 0.624-0.643), 0.688 (95% CI: 0.679-0.696), 0.602 (95% CI: 0.593-0.611), 0.692 (95% CI: 0.684-0.701), and 0.694 (95% CI: 0.686-0.703), respectively (**Figure 6B**). In the independent validation set, the AUROC values of ANN, DT, GBT, K-nn, NB, and RF were 0.713 (95% CI: 0.696-0.730), 0.627 (95% CI: 0.610-0.644), 0.697 (95% CI: 0.679-0.714), 0.593 (95% CI: 0.575-0.612), 0.706 (95% CI: 0.689-0.723), and 0.706 (95% CI: 0.688-0.723), respectively (**Figure 6C**). Details on the model’s performance are shown in **Table 3**. We plotted the predicted model and ideal calibration curves (**Figure 7**) and further evaluated the agreement in terms of calibration slope (an ideal value of 1) and Brier score (an ideal value of 0; a value >0.3 indicates poor calibration). Good calibration was observed for all six machine learning models (**Figures 7A–F**), with Brier scores of 0.182, 0.195, 0.193, 0.229, 0.208, and 0.185, respectively. However, the respective calibration slopes deviated slightly as follows: 1.083, 0.896, 2.769, 0.376, 0.259, and 1.062. Details are shown in **Table 4**.

**Table 3:** Model performance.

Algorithm	Discrimination tests							
	Cutoff	AUROC (95% CI)	Accuracy (95% CI)	Sensitivity (95% CI)	Specificity (95% CI)	Precision (95% CI)	F1-score (95% CI)	KAPPA (95% CI)
ANN	0.710	0.713 (0.696-0.730)	0.669 (0.669-0.669)	0.682 (0.699-0.665)	0.637 (0.665-0.610)	0.822 (0.837-0.807)	0.745 (0.729-0.762)	0.284 (0.313-0.255)
DT	0.765	0.627 (0.610-0.644)	0.688 (0.688-0.688)	0.780 (0.795-0.765)	0.463 (0.491-0.435)	0.781 (0.796-0.766)	0.780 (0.765-0.795)	0.243 (0.274-0.211)
GBT	0.718	0.697 (0.679-0.714)	0.617 (0.617-0.617)	0.576 (0.594-0.558)	0.719 (0.745-0.694)	0.835 (0.851-0.818)	0.682 (0.663-0.700)	0.241 (0.267-0.214)
K-nn	0.590	0.593 (0.575-0.612)	0.659 (0.659-0.659)	0.778 (0.793-0.763)	0.367 (0.394-0.339)	0.751 (0.767-0.736)	0.764 (0.749-0.780)	0.149 (0.180-0.117)
NB	0.802	0.706 (0.689-0.723)	0.669 (0.670-0.669)	0.680 (0.697-0.664)	0.642 (0.670-0.615)	0.824 (0.839-0.809)	0.745 (0.729-0.761)	0.286 (0.315-0.257)
RF	0.718	0.706 (0.688-0.723)	0.652 (0.652-0.652)	0.649 (0.667-0.632)	0.658 (0.685-0.631)	0.824 (0.839-0.808)	0.726 (0.709-0.743)	0.267 (0.295-0.238)

ANN, artificial neural network; DT, decision tree; GBT, gradient-boosting tree; K-nn, k-nearest neighbor; NB, naïve Bayes; RF, random forest.

**Table 4:** Results of calibration curve analysis of six machine learning models for predicting sleep quality in college students.

Algorithm	Calibration		
	Brier score	Slope	Intercept
ANN	0.182	1.083	-0.119
DT	0.195	0.896	0.093
GBT	0.193	2.769	-1.533
K-nn	0.229	0.376	0.682
NB	0.208	0.259	0.648
RF	0.185	1.062	-0.046

ANN, artificial neural network; DT, decision tree; GBT, gradient-boosting tree; K-nn, k-nearest neighbor; NB, naïve Bayes; RF, random forest.

**Figure 8:**
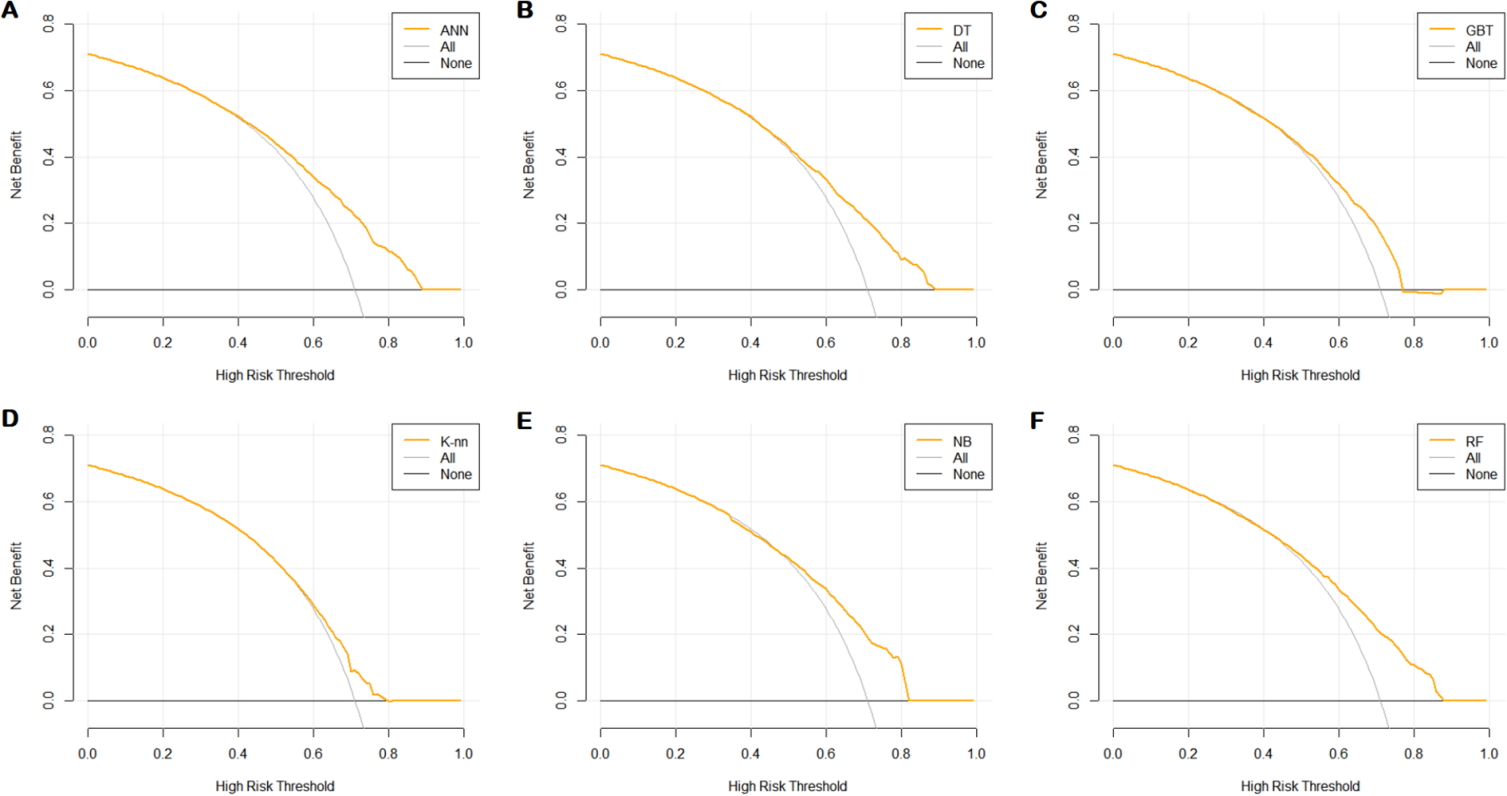
Decision curve analysis of six prediction models. **(A)** ANN; **(B)** DT; **(C)** GBT; **(D)** K-nn; **(E)** NB; **(F)** RF.

In order to determine the clinical usefulness of the models, a decision curve analysis and a clinical impact curve analysis were performed on the prediction models. The clinical decision curves (**Figure 8**) showed that when the clinical decisions were performed using the ANN, DT, GBT, K-nn, NB, and RF prediction models, the threshold probabilities of achieving a greater net benefit than the “no treatment” or “all treatment” scenarios were 0.89, 0.89, 0.88, 0.81, 0.82, and 0.88, respectively.

**Figure 9:**
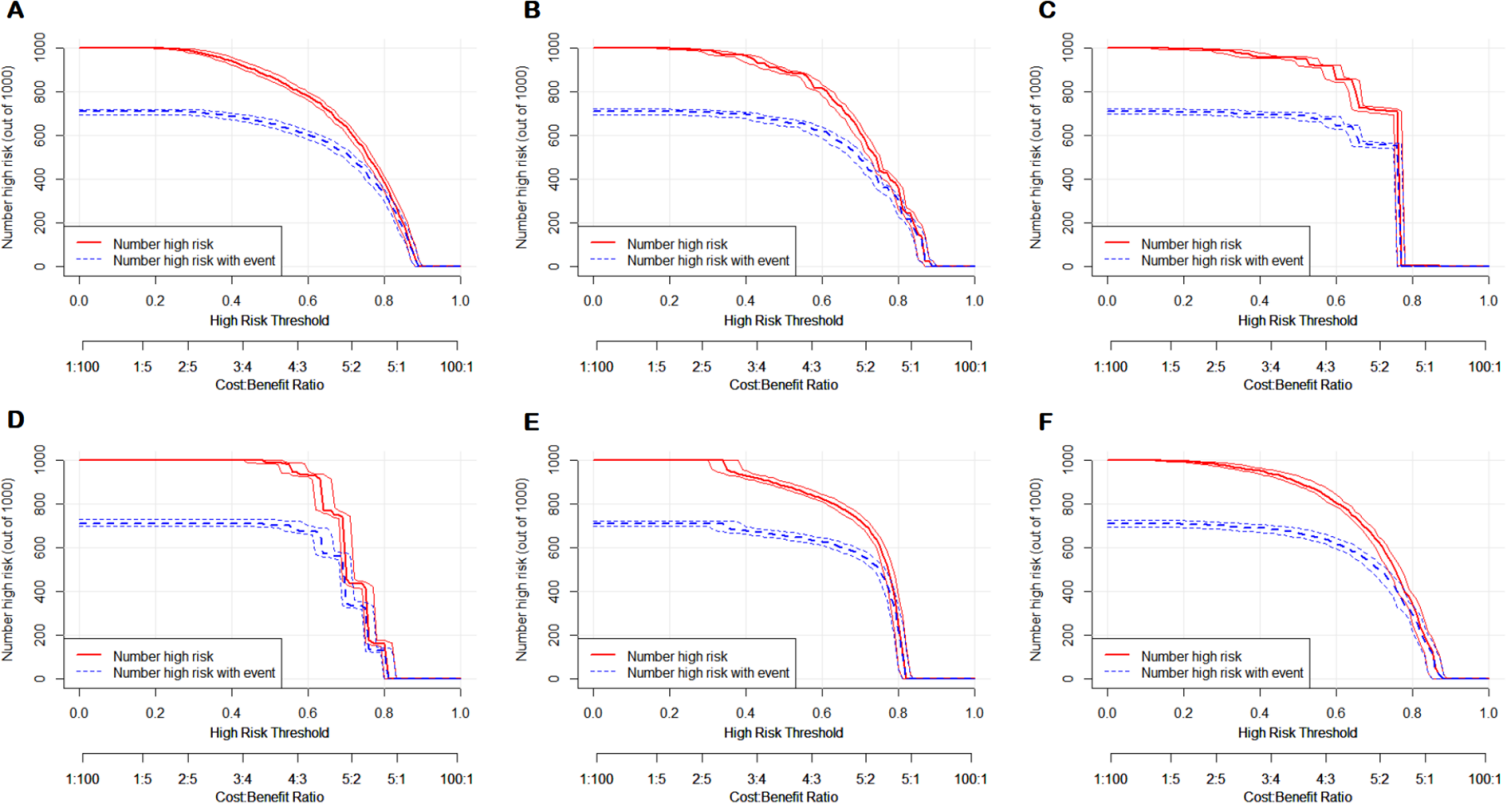
Clinical impact curve analysis of six prediction models. **(A)** ANN; **(B)** DT; **(C)** GBT; **(D)** K-nn; **(E)** NB; **(F)** RF.

A clinical impact curve analysis (**Figure 9**) showed the clinical effectiveness of the six predictive models. The ANN, DT, GBT, K-nn, NB, and RF models were judged to be a high match between those with poor sleep quality and those with actual poor sleep quality when the threshold probabilities were greater than 75%, 70%, 75%, 65%, 70%, and 75%, respectively, confirming the high clinical efficiency of the prediction model.

### Clinical applications

Clinically, this model can provide actionable insights for assessing sleep-related risks and informing intervention measures. By inputting participant-specific information—such as age, specialty, respiratory history, coffee consumption, late-night habits, prolonged online activity, sudden life changes, and impatient closed-loop management—the model generates a risk score for poor sleep quality ranging from 0% to 100%. Using a threshold of 50%, the model offers tailored recommendations. If the risk score is ≥50%, further diagnostic evaluation and targeted treatment are advised. If the risk score is <50%, lifestyle modifications and regular follow-ups are recommended. To enhance accessibility and practical application, we have developed a user-friendly website (cosleep.angelong.cn) that integrates the ANN model. This platform allows both participants and clinicians to input relevant predictors and obtain immediate risk assessments along with actionable advice.

## Discussion

### Main findings

The current study is a valuable addition to the field as it created and verified a prediction model for estimating sleep quality. This model is easily accessible due to the utilization of the following eight readily available variables: age, specialty, respiratory history, coffee consumption, stay up, long hours online, sudden changes, and impatient closed-loop management.

During the COVID-19 pandemic, the contagious nature and uncertainty surrounding the virus have heightened fear of infection, leading to increased psychological stress and anxiety, which in turn adversely affected their sleep patterns. The implementation of closed-loop management significantly restricted students’ mobility, limiting their ability to move freely on campus, participate in extracurricular activities, or engage in leisure activities as they previously could. Such restrictions contributed to feelings of depression and irritability among students. Prolonged exposure to these emotional states has been shown to negatively impact sleep quality. In a study conducted by Kwon, Mihyoung et al., data analysis revealed that fear of COVID-19 is a significant factor influencing sleep quality, with a strong positive correlation observed between COVID-19-related fear and declines in sleep quality. These findings align closely with our conclusions (34).

Additionally, our results showed good predictive ability of our fitted models (i.e., cutoff, AUROC, accuracy, sensitivity, specificity, precision, F1-score, and KAPPA values of 0.710, 0.713, 0.669, 0.682, 0.637, 0.822, 0.745, and 0.284, respectively). In addition, the Brier score was 0.182. The calibration curves showed good agreement between the predictions and the observations. The decision curve analysis demonstrated that the model could achieve a net benefit. The clinical impact curve confirmed the high clinical efficiency of the prediction model.

To evaluate whether we could gain a sufficient sample size to draw conclusions, we performed a *post hoc* sample size calculation based on an online interactive tool (https://riskcalc.org/samplesize/). In the final model with eight predictors, we used the C-statistic in conjunction with the expected incidence to approximate the Cox–Snell R-squared and found that the poor sleep quality incidence was 28.9% for all participants. A minimal sample of 316 participants and a minimum of 11.42 events per predictor parameter were required. Thus, the actual sample of 20,645 patients in this study likely provided sufficient power to ensure the reliability of our results.

### Strengths

To the best of our knowledge, this is the first prediction model derived from a LASSO algorithm for sleep prediction aimed at college students.

**Figure 10:**
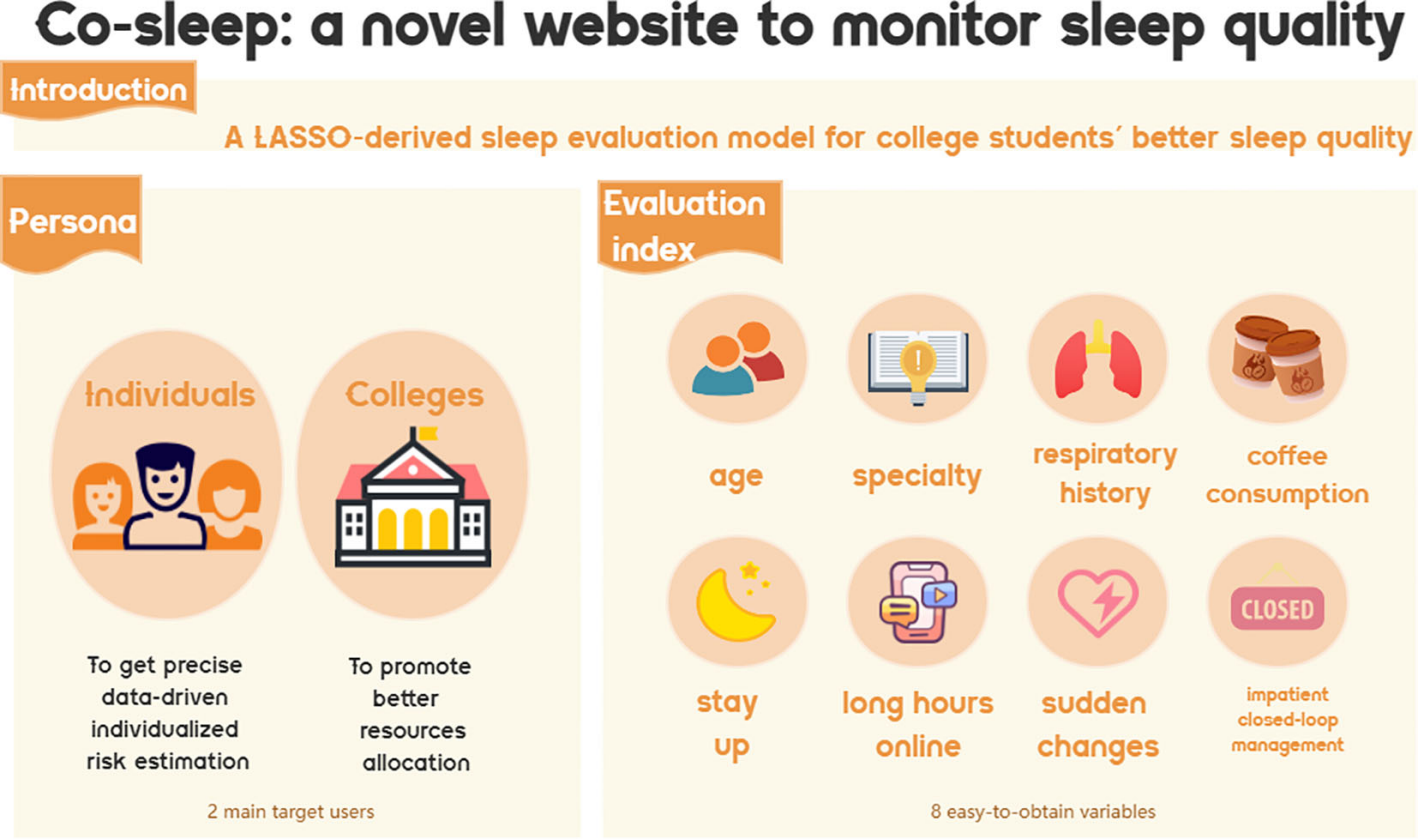
The model was used to establish a LASSO-derived sleep evaluation website for better sleep quality among college students.

Our team has made a significant breakthrough by creating an intuitive website (cosleep.angelong.cn) that allows students and administrators to effectively monitor their sleep quality in comparison to a previous study (26). This innovative platform will provide valuable insights into sleep patterns and ultimately improve overall wellness. It could boost more precise, data-driven, individualized risk estimations and promote better healthcare resource allocations. A novel insight into this application is shown in **Figure 10**.

This study is superior to a previous study (26) because here we updated the algorithm to select the eight most significant variables using LASSO and developed and validated a model with additional evaluation metrics (i.e., cutoff, F1-score, Brier score, decision curve analysis, and impact curve analysis).

Compared with other studies, Kim B.J. et al. used OSA and obesity as predictors to assess sleep quality using logistics, which lacked certain clinical predictive efficacy and generalizability (35). Lang, C. et al. studied sleep quality in adolescents by combining subjective and objective approaches using physical activity as a predictor (36), and Qing Hai Gong et al. used the dietary behaviors of adolescents as predictors. However, the above indicators are difficult to obtain and record and do not have advantages in large-scale predictions (37).

The preliminary identification of variables for the final modeling using a multinomial logistic regression, LASSO regression, and Boruta feature selection was less dependent on the researcher’s intuition. The use of machine learning (i.e., ANN, DT, GBT, K-nn, NB, and RF models) was also possible because large-scale representative data was used. In this study, the LASSO algorithm was used to select eight easy-to-obtain predictors and combined with machine learning algorithms to build a prediction model. Additionally, a *post hoc* sample size analysis based on an interactive online website showed good clinical prediction efficacy and generalizability of the model.

### Limitations

There were several limitations to this study. First, since a cross-sectional design was used, the shortcomings associated with this design could not be avoided. Cross-sectional studies only reflect a situation at a particular point in time, but due to their shortcomings in causal co-occurrence research, they cannot determine the causal relationship between sleep quality and factors such as psychological conditions. Second, since these college students were not independently sampled, some bias might have been introduced in the sampling process. Third, the data were derived from self-assessments via online surveys, which inevitably introduced some instability in the results. Fourth, according to the adherence survey, fewer students were willing to undergo evening sleep quality monitoring (i.e., use wearable devices or smart bracelets) and post-follow-ups, making it challenging to conduct further cohort studies. This could also pose difficulties to conduct further assessment and analysis of their sleep quality at different times. Fifth, although our machine learning modeling appeared to have good predictive ability, the results are dependent on the data used in the development and validation stages. If possible, external validation of college students from other provinces in China should be performed to produce a better-trained prediction model. Sixth, the adaptability of the findings may face challenges when applied to diverse populations. The core characteristic variables in the model—such as age, major, coffee intake, and staying up late habits—primarily reflect the lifestyle and environmental characteristics of college students in Fujian, China. However, factors influencing sleep patterns can vary significantly across different demographic groups (e.g., medical students vs. non-medical students, adolescents with vs. without a family psychiatric history, graduate vs. undergraduate students) (19, 38, 39). Furthermore, certain variables included in the model—such as “impatience with closed-loop management”—are closely tied to the specific pandemic-related management practices of Chinese universities, which may not be applicable to other regions or time periods. Additionally, cultural differences in behaviors such as coffee consumption and staying up late habits (e.g., higher coffee intake habits in international students from Europe or America) could further limit the predictive validity of the model when applied to populations outside the study context.

### Future directions

In terms of further research, we will explore the performance of this model in other regions and utilize wearable devices to track the sleep quality of college students continuously. We would not just estimate the risk factors for sleep disturbance but also transform them into later interventions. The published literature on early interventions among the university student population with no prior sleep-related pathologies is scarce, despite the fact that they are considered a high-risk group (40–43). Once we identify students with high risk of sleep disturbance, we can study different early interventions (e.g., pharmacological intervention, behavioral sleep-promoting intervention, or no intervention) and quantify the effects of different intervention types on the sleep characteristics of adolescents and emerging adults who do or do not have a sleep disorder by studying the outcomes, including changes in the sleep measure scores that represent at least one of the key sleep metrics such as total sleep time (TST), sleep efficiency (SE), wake after sleep onset (WASO), and sleep onset latency (SOL) measured with actigraphy/polysomnography (PSG), sleep stages (rapid eye movement sleep and non-rapid eye movement sleep [stages 1, 2, and 3], as evaluated with PSG only). Furthermore, we recommend that future studies incorporate accelerometers to objectively assess physical activity levels. This approach would significantly improve the validity and reliability of the findings by reducing reliance on self-reported data and providing more accurate measurements of both sleep and activity patterns (36).

Additionally, we can combine some relevant psychological scales, such as Beck Depression Inventory (BDI) (44), Kessler Psychological Distress Scale (K6) (45), Kessler Psychological Distress Scale (K10) (46), Fatigue Scale 14 (FS-14) (47), Generalized Anxiety Disorder Scale (GAD-7) (48) and Eating Attitudes Test (EAT-26) (49) to identify other significant variables from different perspective.

While our research focused on college students in Fujian Province, China, we acknowledge that cultural, academic, and lifestyle differences across regions may impact the model’s performance in other settings. To address this, we plan to conduct external validation using our an intuitive website (cosleep.angelong.cn) in other provinces in China and, if possible, from international cohorts. This will allow us to assess the model’s robustness and adaptability across diverse cultural and educational contexts.

### Implications

In our previous study, it was found that a student’s residence significantly affected their quality of sleep. However, the previous study’s limitations highlighted the need to confirm the effectiveness of residence as a predictor in the model (26). In the current study, the LASSO regression analysis did not identify residence as a significant variable. This implied that the residence factor might not have a decisive impact on the sleep quality of college students. Therefore, this supported the use and popularization of this model at a broader geographical range of universities.

Undiagnosed sleep problems can worsen the mental stress experienced by college students, potentially leading to long-term health consequences for both the individuals and the healthcare system (50). By examining the sleep quality of college students in the post-epidemic era, we can identify those at high risk for sleep problems and design targeted health promotion interventions that address modifiable factors. This study aimed to identify significant variables associated with poor sleep quality and use them to create an easy-to-use website. The website accurately evaluated the probability of suffering from poor sleep quality, particularly among university students. Improving the sleep quality of college students through early interventions can lead to increased awareness of sleep health and, ultimately, better wellbeing and academic performance. By predicting sleep quality and implementing interventions, we can promote universal education on sleep health among college students. Ultimately, optimizing sleep health will benefit the overall wellbeing and academic success of college students.

## Conclusion

The prediction model, which incorporated eight predictors, was built using a LASSO regression and an ANN to estimate the probability of sleep disturbance among college students. Additionally, based on this model, we built an easy-to-operate website (cosleep.angelong.cn) for improved monitoring, which may be used as an intuitive and practical tool by both individuals and school management.

## Data Availability

The source code and the datasets used in this study are freely available at https://github.com/ChunmeiFan/PSQI-Prediction.git.

## Supplementary material

Supplementary Text S1 A Survey on the Sleep Quality of College Students during COVID-19 Epidemic in Fujian Province.
