# Supplementary material for: Novel insight into prediction model for sleep quality among college students: a LASSO-derived sleep evaluation
Source: Front Psychiatry. 2025 Apr 25;16:1585732. doi: 10.3389/fpsyt.2025.1585732 (PMC12061895; doi:10.3389/fpsyt.2025.1585732)
Supplement: Supplementary Text S1 — A Survey on the Sleep Quality of College Students during COVID-19 Epidemic in Fujian Province. [file Table1.docx]

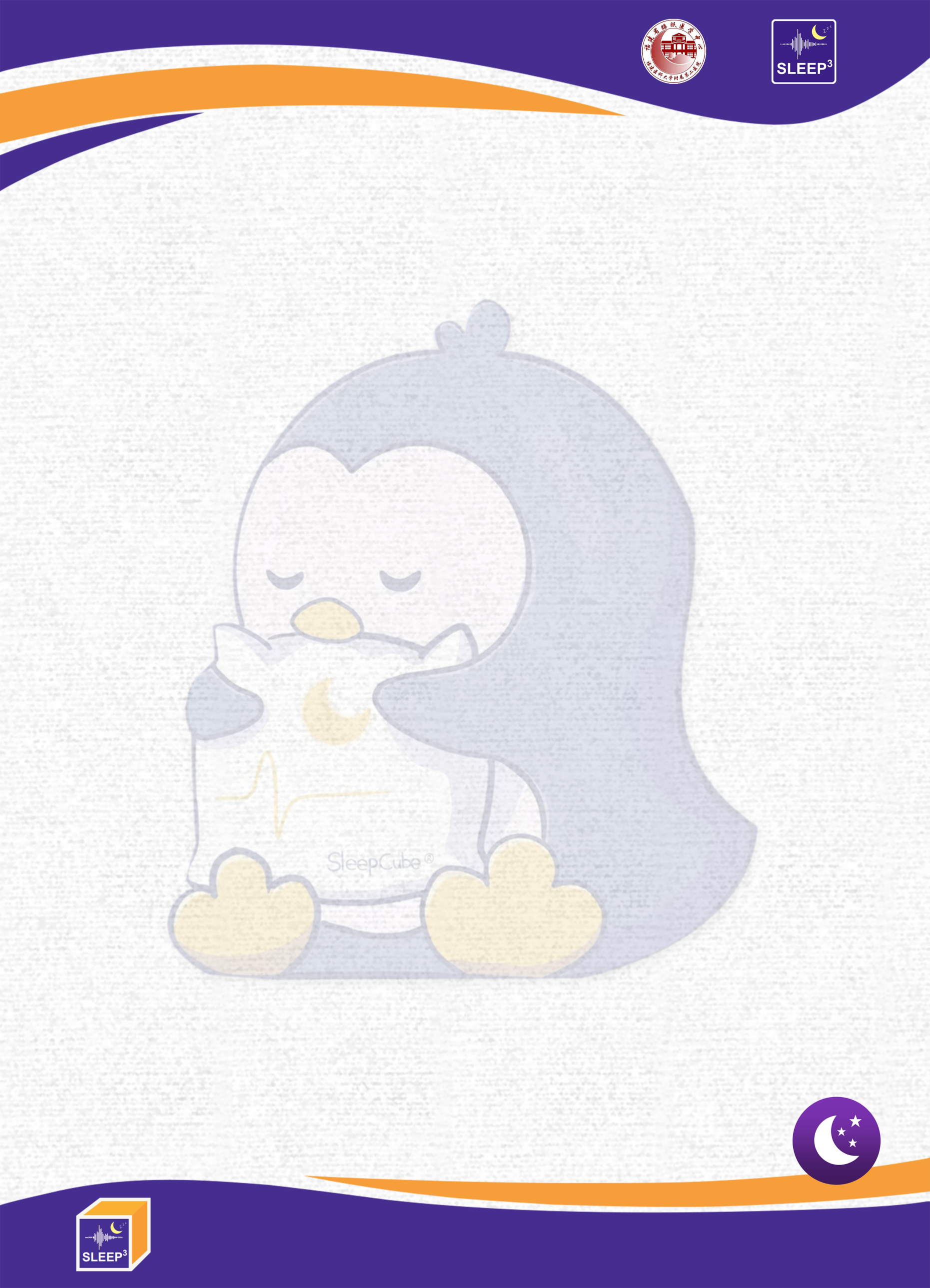


A Survey on the Sleep Quality of College Students during COVID-19 Epidemic in Fujian Province

Dear students,

We are the investigators of the "A Survey on the Sleep Quality of College Students during COVID-19 Epidemic in Fujian Province". We are from Fujian Medical University and Fujian Sleep Medicine Center. Due to the severe situation of the COVID-19 epidemic from March to April 2022, Fujian Province has implemented strict control measures in universities, including restricting access to campus and shifting offline courses to online. These measures have significantly changed the normal study and life patterns of college students.

In this survey, our research team aims to explore and evaluate the sleep quality of college students in Fujian Province during the COVID-19 period. We also aim to explore the factors that influence their sleep quality and provide insights for improving their sleep quality. The questionnaire includes general data collection, the Pittsburgh Sleep Quality Index (PSQI), and factors affecting sleep quality.

We sincerely hope to have your support and participation in this survey. Your honest answers will be highly appreciated. Please be assured that all personal information collected in this survey will be strictly confidential and will only be used for this research project. Thank you for your support.

Best regards,

Key Laboratory of Sleep Medicine in Fujian Province, Fujian Sleep Medicine Center

Enclosure: Questionaire of sleep quality

***Sleep Well Live Well***

Questionaire of sleep quality

Enclosure-1

**
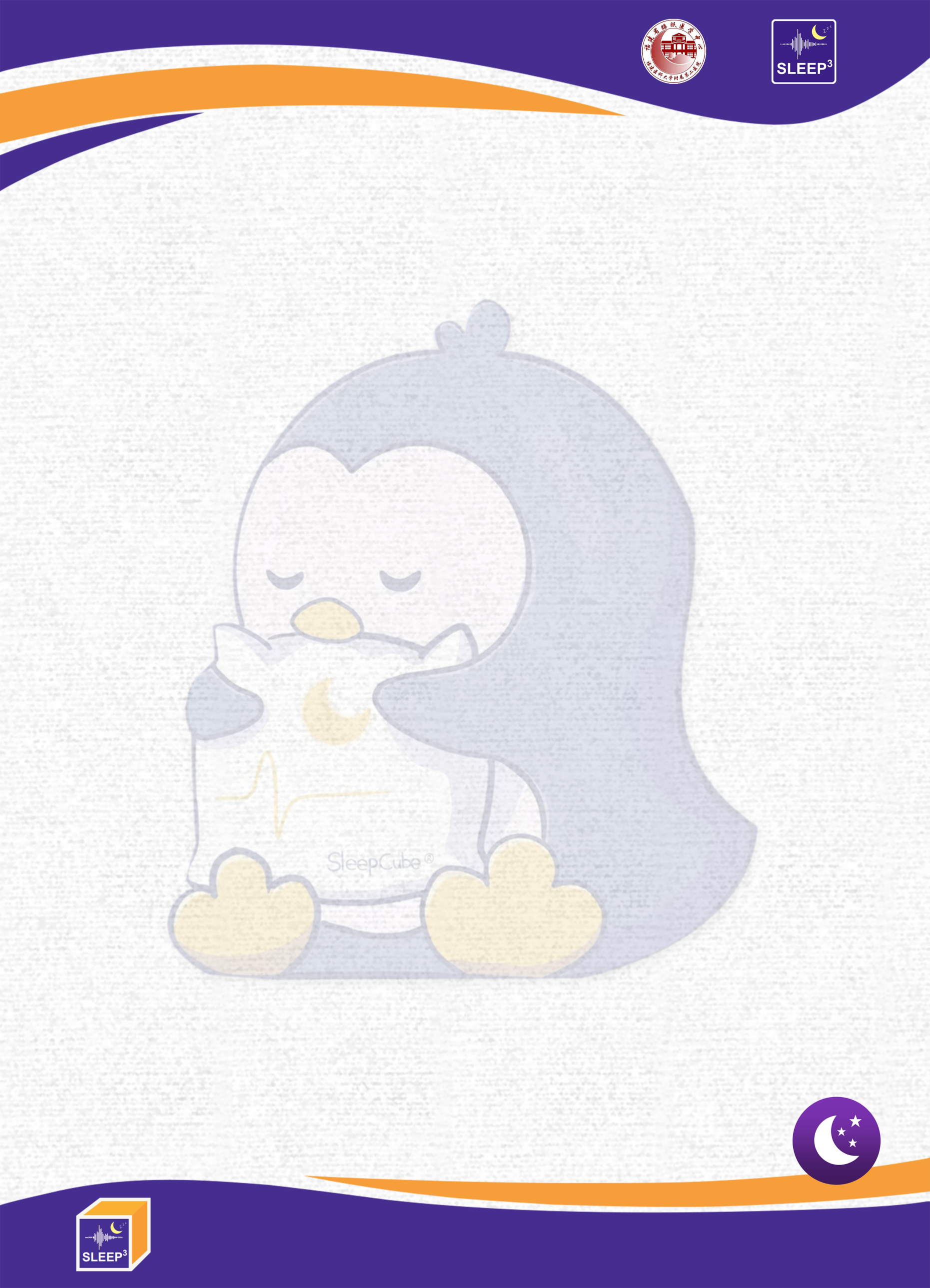
Part 1 General data collection**

***Sleep Well Live Well***

1. Gender: A. Male B. Female
2. Age: ____
3. School: ____
4. Major: A. Medical related majors B. Science and engineering majors C. Liberal arts
5. Current situation: A. Graduation class B. Non graduation class
6. Height (cm): ____
7. Weight (kg): ____

**Part 2 Pittsburgh Sleep Quality Index (PSQI)**

**[Please fill in the following questions based on the actual situation in the past month]**

1. In the past month, the usual time to go to bed at night is ____ o'clock.
2. In the past month, usually wake up at ____ o'clock.
3. In the past month, it usually takes ____ minutes from going to bed to falling asleep.

A. ≤ 15 minutes B. 16-30 minutes C. 31-60 minutes D. ≥ 60 minutes

4. In the past month, the actual sleep time per night is usually _____ hours (This may be different than the number of hours you spend in bed).

A. >7 hours B. 6-7 hours C. 5-6 hours D. <5 hours

5. Difficulty falling asleep in the past month (unable to fall asleep within 30 minutes)

A. No B. Less than once a week C. Once or twice a week D. Three or more times a week

6. In the past month, wake up in the middle of the night or early morning

A. No B. Less than once a week C. Once or twice a week D. Three or more times a week

7. In the past month, go to the bathroom at night

A. No B. Less than once a week C. Once or twice a week D. Three or more times a week

8. In the past month, cannot breathe comfortably

A. No B. Less than once a week C. Once or twice a week D. Three or more times a week

9. In the past month, cough or snore loudly

A. No B. Less than once a week C. Once or twice a week D. Three or more times a week

10. In the past month, feel too cold

A. No B. Less than once a week C. Once or twice a week D. Three or more times a week

11. In the past month, feel too hot

A. No B. Less than once a week C. Once or twice a week D. Three or more times a week

12. In the past month, nightmares
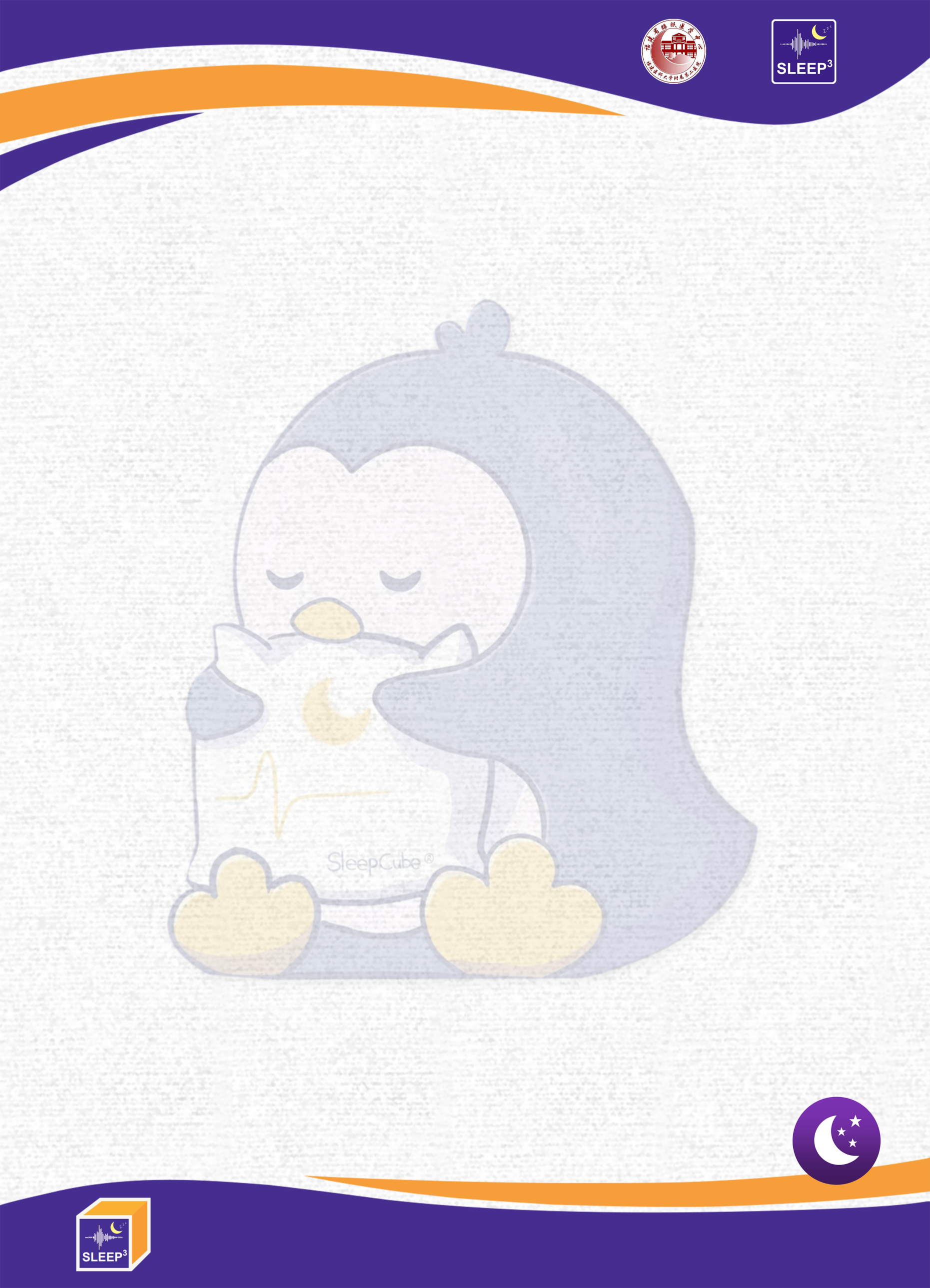


***Sleep Well Live Well***

Enclosure-2

A. No B. Less than once a week C. Once or twice a week D. Three or more times a week

13. In the past month, pain and discomfort

A. No B. Less than once a week C. Once or twice a week D. Three or more times a week

14. Other reason(s) that affect sleep in the past month

A. No B. Less than once a week C. Once or twice a week D. Three or more times a week

15. In the past month, how would you rate your sleep quality overall

A. Very good B. Fairly good C. Fairly bad D. Very bad

16. In the past month, how often have you taken medicine (prescribed or “over the counter”) to help you sleep

A. No B. Less than once a week C. Once or twice a week D. Three or more times a week

17. In the past month, Have you often felt drowsy in the past month

A. No B. Less than once a week C. Once or twice a week D. Three or more times a week

18. Have you had insufficient energy to work in the past month

A. No B. Occasionally C. Sometimes D. Often

**Part 3 Factors Affecting Sleep Quality**

1. Do you have any major sleep disorders

A. Yes B. No

2. Do you have any major mental disorders?

A. Yes B. No, but with family history C. No, and without family history

3. Do you suffer from respiratory diseases such as rhinitis?

A. Yes B. No

4. Do you never sleep, and are you physically healthy and energetic?

A. Not sure B. Sometimes C. Often D. Always

5. Do you have a habit of consuming caffeinated beverages on a daily basis (such as milk tea or coffee)?

A. No B. Occasionally C. Frequently D. Almost every day

6. After the school implemented closed-loop management, did you still maintain your previous dietary routine?

A. Not sure B. Sometimes C. Often D. Always

7. After the school adopts closed-loop management, do you prefer to stay up late to complete learnin
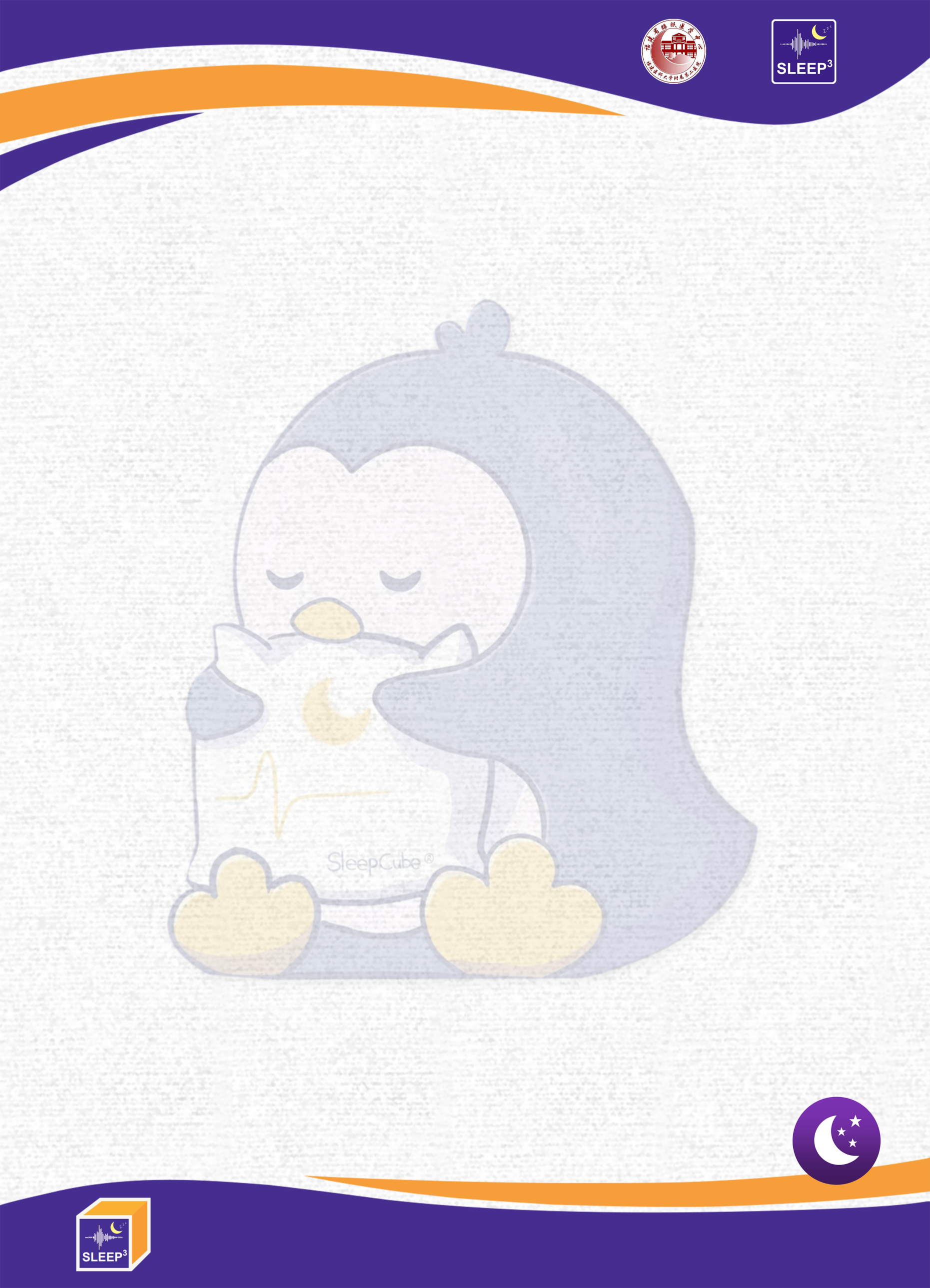
g work?

Enclosure-3

Enclosure-3

A. Not sure B. Sometimes C. Often D. Always

***Sleep Well Live Well***

Enclosure-4

8. After closed-loop management, did you extend your time using electronic products for entertainment and relaxation (not online learning time)?

A. Not sure B. Sometimes C. Often D. Always

9.Have you encountered any severe events in your life during the past month?

A. Yes B. No

10. Are you worried that yourself, your relatives, your classmates will be infected with COVID-19?

A. No B. Sometimes C. Often D. Always

11. Are you feeling irritable and depressed due to restrictions on going out?

A. Not sure B. Sometimes C. Often D. Always


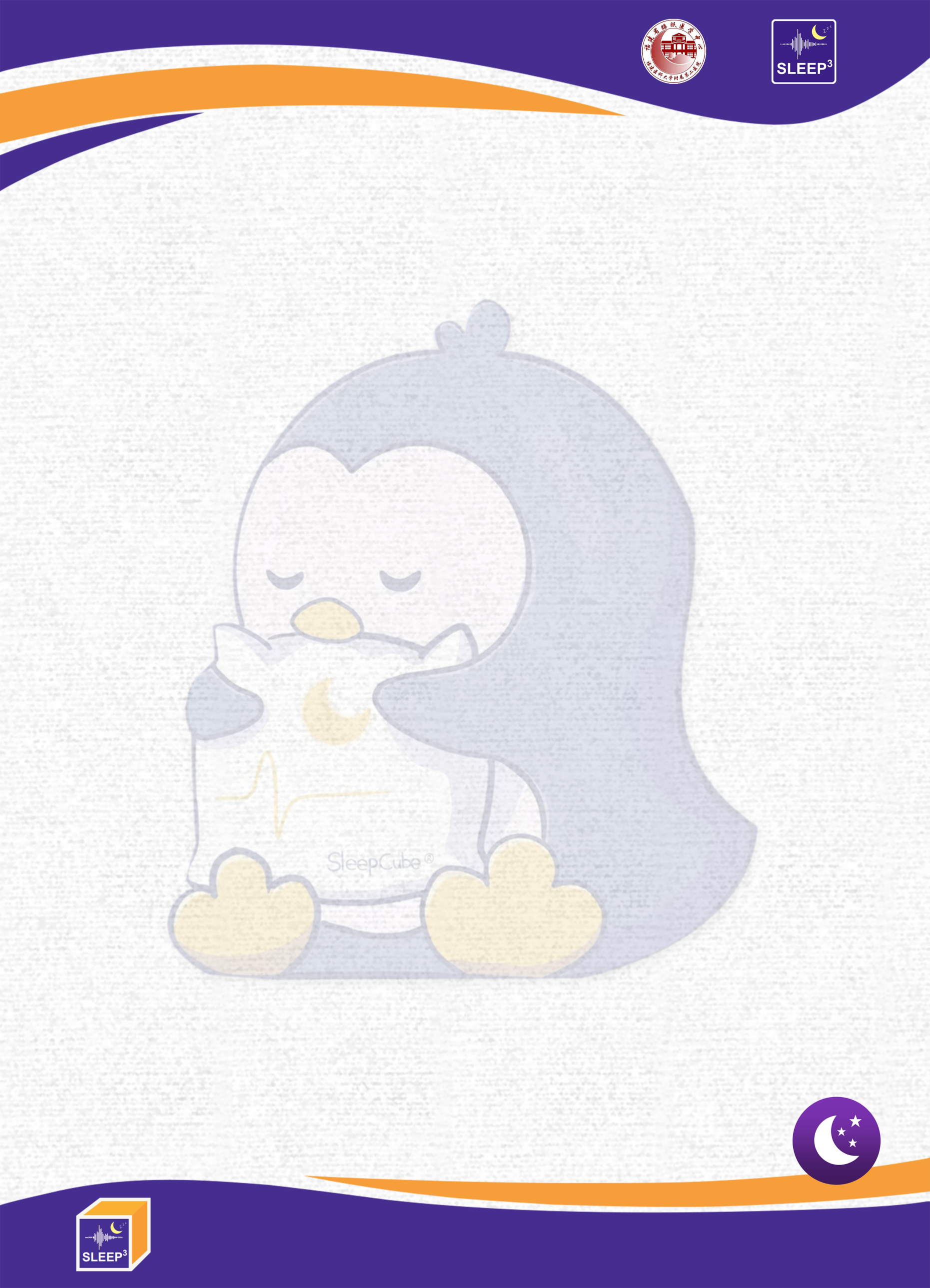


***Sleep Well Live Well***

Enclosure-4

***Sleep Well Live Well***
